# Extracellular vesicles in Niemann pick disease type C: current knowledge and future opportunities

**DOI:** 10.3389/fncel.2025.1730603

**Published:** 2026-01-22

**Authors:** Sarah Catherine B. Hawthorne, Ursula S. Sandau, Julie A. Saugstad

**Affiliations:** Department of Anesthesiology and Perioperative Medicine, Oregon Health & Science University, Portland, OR, United States

**Keywords:** endolysosomal pathway, extracellular vesicles, lysosomal storage disorder, neurodegeneration, Niemann pick disease type C, rare disease

## Abstract

Niemann Pick Disease Type C (NPC) is a rare neurodegenerative disease that primarily affects children. It is caused by mutations in the *NPC1* or *NPC2* genes, which encode proteins that transport cholesterol out of the endolysosomal organelles. Endolysosomal compartments also produce extracellular vesicles (EVs), which have emerged as key players in human disease. There is rapidly growing interest in how NPC cellular pathology impacts EV biology: of the 18 peer-reviewed publications on this topic, 13 were published within the last 5 years. Collectively, the existing literature suggests that the NPC proteins play key roles in EV biogenesis and uptake, that EV concentration and cargo are fundamentally altered in samples with NPC1/2 protein dysfunction, and that EVs may contribute to the therapeutic effects of NPC treatments. To better elucidate the connections between NPC and EVs further research is needed, especially in patient samples. Ultimately, a better understanding of the role of EVs in NPC will likely shed light on basic EV biology, related cellular neuropathologies, and a rare childhood disease that currently has no cure.

## 1 Introduction

Niemann Pick Disease Type C (NPC) is a rare autosomal recessive disorder characterized by lipid accumulation and neurodegeneration (Vanier, 2010). NPC primarily affects infants, children, and young adults, and has an estimated prevalence of 1 in every 100,000 births (Berry-Kravis, 2021). Average life expectancy for NPC patients is 13 years of age, although there is high heterogeneity in age of onset and disease progression (Bianconi et al., 2019). In 2024 the U.S. Food & Drug Administration approved its first two treatments for NPC, representing an exciting breakthrough for researchers and patients: Miplyffa (arimoclomol) was approved to treat NPC in adults and children aged 2 years and older, and Aqneursa (N-acetyl-L-leucine) was authorized to address neurological symptoms associated with NPC in both adult and pediatric patients weighing at least 15 kg (Jan et al., 2025). However, both drugs are aimed at managing symptoms, and the search for curative treatments continues.

NPC is neurovisceral disorder that causes symptoms in both the central nervous system (CNS) and peripheral organs, and clinical manifestations of NPC are typically age dependent. In infancy and early childhood, NPC primarily presents as a liver and spleen disease accompanied by subtle neurological symptoms, such as delayed developmental milestones (Geberhiwot et al., 2018). Adolescent and adult patients are more likely to present with psychiatric symptoms that can mimic schizophrenia or mood disorders (Rego et al., 2019). Neurodegenerative symptoms including motor control issues (clumsiness, seizures, gaze palsy), behavioral changes, and memory loss are common in patients of all ages (Berry-Kravis, 2021), and nearly 70% of patients experience cognitive decline at a high enough severity to meet criteria for a dementia diagnosis (Rego et al., 2019). Indeed, NPC is often called childhood Alzheimer’s due to its similarities with the pathology and clinical symptoms of age-related Alzheimer’s disease (AD)‑ (Malnar et al., 2014). However, NPC is an extremely heterogeneous disease resulting from an array of genetic mutations, and significant variation in age of disease onset, types of symptoms, and rate of progression has been observed between patients.

Across patients of all ages, the cellular hallmark of NPC is abnormal cholesterol accumulation within late endosomes and lysosomes and consequent disruption of the endolysosomal pathway (Vanier, 2010). This pathway is also responsible for producing extracellular vesicles (EVs). EVs are membranous nanoparticles that are produced by all cells and can be taken up by distinct recipient cells, allowing them to play important roles in cell-to-cell communication. EV molecular cargo can include proteins, lipids, nucleic acids, and metabolites from the parent cell. EV cargo is influenced by disease state and accordingly, EVs can play significant roles in pathology and serve as biomarkers for both lipid storage disorders (Eitan et al., 2016; Gleason et al., 2021) and neurodegenerative diseases (Rastogi et al., 2021; Soliman et al., 2021). Interestingly, many elements of EV biology are likely impacted by the various cellular disruptions that occur in NPC (Wheeler and Sillence, 2020). However, little is known about how mutations in the NPC proteins affect EVs.

In this review, we will examine both NPC and EV biology and discuss our current understanding of the role of EVs in NPC. Throughout, our focus is to highlight current understanding and opportunities for further research. We note that of the 18 peer-reviewed articles that directly examine EVs in the context of NPC, 13 were published in the last 5 years indicating growing scientific interest and the opportunity to uncover both novel disease biology and therapeutic targets.

## 2 NPC cellular pathology

NPC is caused by mutations in either the *NPC1* gene (95% of cases) or the *NPC2* gene (5% of cases), which, respectively, code for the NPC1 and NPC2 proteins (Vanier, 2010). Over 400 pathogenic mutations in *NPC1* and *NPC2* have been identified (Mavridou et al., 2017). The most common *NPC1* mutation (I1061T) is found in 20% of patients, while the remaining 80% have mutations that are often unique or shared among only a handful of other patients (Millat et al., 1999). To add further complexity, NPC patients are often compound heterozygotes, meaning that they inherit a different disease-causing allele from each parent and thus have two different *NPC* mutations. This genetic variety likely contributes to the observed heterogeneity in patient disease onset and progression, as each mutation impedes the function of the NPC1 or NPC2 proteins to a different extent. The high genetic and clinical heterogeneity is also a key challenge to understanding NPC biology, as studies that focus on a single mutation or use genetic knockouts generate results that may only be relevant to a small proportion of NPC patients. Building *in vitro* or *in vivo* models that represent the full spectrum of NPC patients is challenging, and given the rarity of NPC, human studies struggle to obtain truly representative sample sizes.

**Figure 1:**
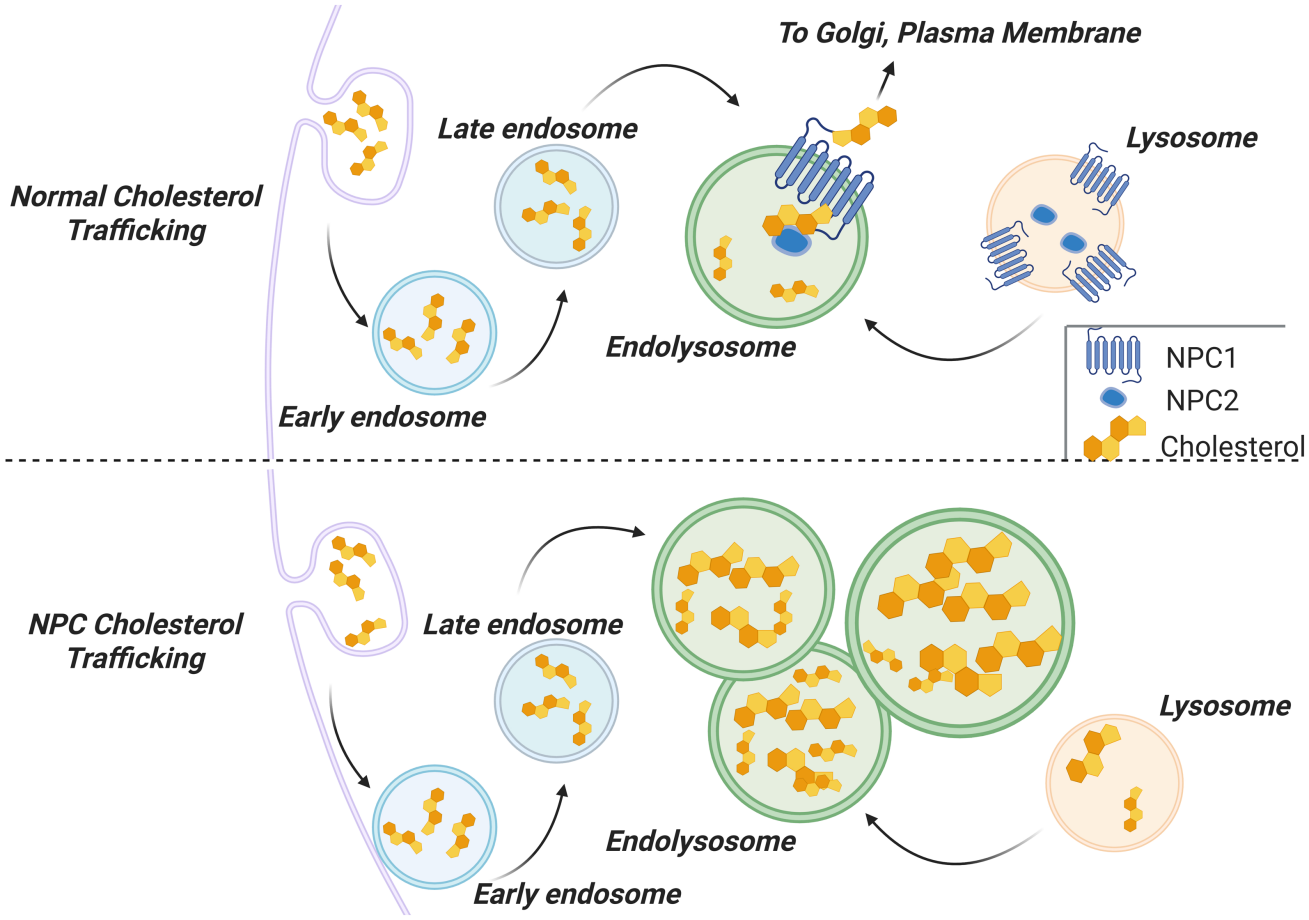
Cholesterol trafficking under normal (**top**) and NPC disease (**bottom**) conditions. Cholesterol enters the cell through endocytosis. Early endosomes mature into late endosomes and fuse with lysosomes to produce endolysosomes. NPC1 and NPC2 proteins then move cholesterol across the endolysosomal membrane, allowing it to enter the cytoplasm and be further trafficked to the Golgi apparatus or plasma membrane. In NPC disease, loss of NPC1 or NPC2 results in cholesterol becoming trapped in endolysosomes, causing them to enlarge and multiply. The rest of the cell enters cholesterol starvation.

Both *NPC1* and *NPC2* are evolutionarily conserved and highly expressed in all human cells (Carstea et al., 1997). NPC1 is a large protein (1,280 amino acids) that resides in the endolysosomal membrane, while the smaller NPC2 protein (130 amino acids) is localized to the endolysosomal lumen (Vanier and Millat, 2004). The role of NPC1 and NPC2 is to transport cholesterol out of endolysosomes (Figure 1). Following endocytosis, cholesterol resides in early endosomes; these organelles then mature into late endosomes and finally merge with lysosomes to form endolysosomes. In the endolysosomal lumen NPC2 binds cholesterol at its sterol-sensing domain. The NPC2/cholesterol complex then binds to NPC1, which has both cholesterol and NPC2 binding domains on its luminal side (Trinh et al., 2018). NPC1 then transports cholesterol across the endolysosomal membrane and into the cytosol, likely by passing the lipid along the sterol-sensing domain embedded in the NPC1 transmembrane domains (Pfeffer, 2019). Mutations in any of the key domains of NPC1 or NPC2 interrupt this pathway, causing endolysosomes to accumulate significant amounts of cholesterol while the rest of the cell enters a state of cholesterol starvation (Bajwa and Azhar, 2021).

Importantly, the connection between disrupted cellular cholesterol trafficking and NPC disease pathology is not fully elucidated, especially regarding alterations in the CNS. The neurobiology of NPC is complex and incompletely understood, but evidence suggests that *NPC1* loss in neurons is a key driver of disease pathology (Lopez et al., 2011). Purkinje neurons of the cerebellum are known to die in an anterior-to-posterior pattern as NPC progresses, and neuronal cells of the frontotemporal cortex and other brain regions can also be affected (Rego et al., 2019). An early study provided evidence in an NPC knockout mouse model that Purkinje cell loss did not proceed by an apoptotic pathway that can be inhibited by Bcl-2 or minocycline, a drug that inhibits apparent apoptotic death in other mouse models of neurological disease (Erickson and Bernard, 2002). Why neuronal cells are particularly sensitive to the loss of the NPC proteins is currently unknown; neurons do not express *NPC1* or *NPC2* at particularly high levels, and patterns of cholesterol accumulation do not correlate with patterns of neuronal death (Martin et al., 2020; Cariati et al., 2021). Some researchers have hypothesized that neurodegeneration in NPC is related to impaired crosstalk between neurons and other cells. Indeed, loss of *NPC1* results in inflammatory responses across brain regions, and corresponding glial defects such as hypomyelination, microgliosis, and reactive astrocytosis (Rego et al., 2019). For instance, early studies identify glial cells as a major target for pathology in a mouse model of NPC, and that a lack of myelin within the corpus callosum may be related to the loss of nerve fibers in this structure (German et al., 2002). Further, the finding of an increase in cathepsin-D-laden microglial cells, in brain regions that undergo neurodegeneration, support that microglia contribute to the neurodegenerative process by phagocytosing dead neurons. And the finding that activation of astrocytes in regions that undergo neurodegeneration was consistent with a role for these glial cells in the neurodegenerative process (German et al., 2002). While each of these could impair neuronal health, the exact mechanism linking loss of *NPC1* and *NPC2* to clinical neurodegenerative symptoms is not well understood.

NPC also causes pathologies with the inner ear, including auditory and balance issues that are common in older NPC patients (Senirli et al., 2016). Early studies to examine patients with NPC1 and hearing loss suggested that multiple steps along the auditory pathway are affected, suggesting that hearing loss is an early and sensitive marker of disease progression (King et al., 2014). One compound that has been evaluated as a treatment for NPC is 2-hydroxypropyl-beta-cyclodextrin (HPβCD). While HPβCD can reduce some debilitating symptoms and extend life span, the therapeutic doses used to treat the disease cause hearing loss by selectively damaging only cochlear outer hair cells (Ding et al., 2021). Earlier studies on the mechanism(s) underlying HPβCD-induced hearing loss found that a prestin-dependent mechanism contributes to HPβCD ototoxicity (Takahashi et al., 2016), that HPβCD-induced outer hair cell death is attributed to the structural role of prestin in maintaining the outer hair cells lateral membrane, rather than its motor function (Zhou et al., 2018), and that cochlear damage by HPβCD is unrelated to reactive oxygen species formation (Lee et al., 2019).

The build-up of endolysosomal cholesterol in NPC also creates a number of other cellular problems that may contribute to disease biology (Wheeler and Sillence, 2020). Cholesterol accumulation severely disrupts the lipid profile of NPC endolysosomes, including a buildup of sphingomyelin, glycosphingolipids, and ceramides that together slow endolysosomal processing and disrupt the organelle’s ability to process cellular waste or transport materials (Newton et al., 2018; Tobias et al., 2019). Mitochondria also become disrupted, showing increased contacts with endolysosomes and resulting in increased reactive oxidative species and cell stress signaling (Vázquez et al., 2012; Torres et al., 2017). Finally the autophagy pathway becomes dysfunctional, causing autophagosomes to accumulate which can eventually result in cell death (Pacheco and Lieberman, 2008). Recently, there has been growing interest in how endolysosomal disruptions in NPC may also affect EVs, which can be produced through the endolysosomal pathway. Though not yet fully understood, emerging evidence suggests that EV concentration and cargo is altered by NPC mutations, making EVs important players in disease pathology.

## 3 EV formation, cargo, and analysis

EVs are small particles that are released from nearly every known cell. Typically 50 to 500 nm in size, EVs are enclosed by a lipid bilayer and contain molecular cargo such as proteins, DNA, coding and noncoding RNA, and metabolites (Thery et al., 2018). While technically accurate, these definitions bely the incredible complexity and opportunity contained in these tiny packages. The cargo carried by EVs reflects their cell of origin but does not perfectly mirror it, and because of this cargo EVs can have dramatic effects when taken up by recipient cells (Sardar Sinha et al., 2018; Upadhya et al., 2020; Van Hoecke et al., 2021a). Although EV science is still an emerging field, EVs have been implicated in nearly every type of human disease, including cancer (Richards et al., 2017; Zhou et al., 2021), infection (Costafreda et al., 2020; Picca et al., 2020), neurodegeneration (Rastogi et al., 2021; Soliman et al., 2021), and, as discussed here, rare diseases such as NPC (Tatiana et al., 2020; Gleason et al., 2021).

Secreted EVs can be found in many biological sources, including cell culture media, biofluids, and tissues (Welsh et al., 2024). EVs collected from patient biofluids can be secreted from any organ and thus have organ-specific effects on diseases. However, it is not always possible to determine the origin of EVs isolated from human biofluids, which complicates interpretation of their cargo. For example, there is great interest in the use of blood EVs as surrogates for cerebrospinal fluid EVs and as biomarkers for neurodegenerative diseases. Yet the results from such studies have not been consistent in their conclusions thus far. For example, L1CAM protein has used for the isolation of brain EVs in blood and the potential diagnosis of AD. While L1CAM protein is highly expressed in brain, it also highly expressed in peripheral organs such as kidney, urinary bladder, and skin.^1^ Thus, some studies using multiple methods have not supported L1CAM as a marker to enrich EVs of neuronal origin (Norman et al., 2021; Kadam et al., 2025). In contrast, a recent study provided evidence from single-vesicle analyses that L1CAM immunoprecipitation supported the existence of L1CAM positive EVs in human blood that also carried multiple neuronal markers GAP43, *β*-III-tubulin and VAMP2, demonstrating that L1CAM can be used as a target for the isolation of neuronal-derived EVs from blood (Nogueras-Ortiz et al., 2024). Thus, the rapid development of combined sensitive methods to assess and validate brain-derived EVs and their cargo in blood provides confidence in the development of non-invasive methods for elucidating the roles of EVs in the mechanisms underlying of brain diseases, and ultimately for the development of non-invasive strategies for the diagnosis and classification of neurodegenerative brain diseases from blood.

The most common methods to separate EVs from other extracellular nanoparticles include ultracentrifugation, size exclusion chromatography, and immunocapture, often in combination, and each method can enrich for slightly different EV populations (Livshits et al., 2015; Konoshenko et al., 2018; Doyle and Wang, 2019; Stam et al., 2021). Methods to analyze EVs may also be biased towards certain EV subpopulations; for example, size-based methods may not be able to detect the smallest EV populations, and methods that include labelling the EV membrane may preferentially dye EVs with certain lipid compositions. Thus, when comparing EV studies, it is important to consider the method used to prepare the EV samples and downstream analytics.

**Figure 2:**
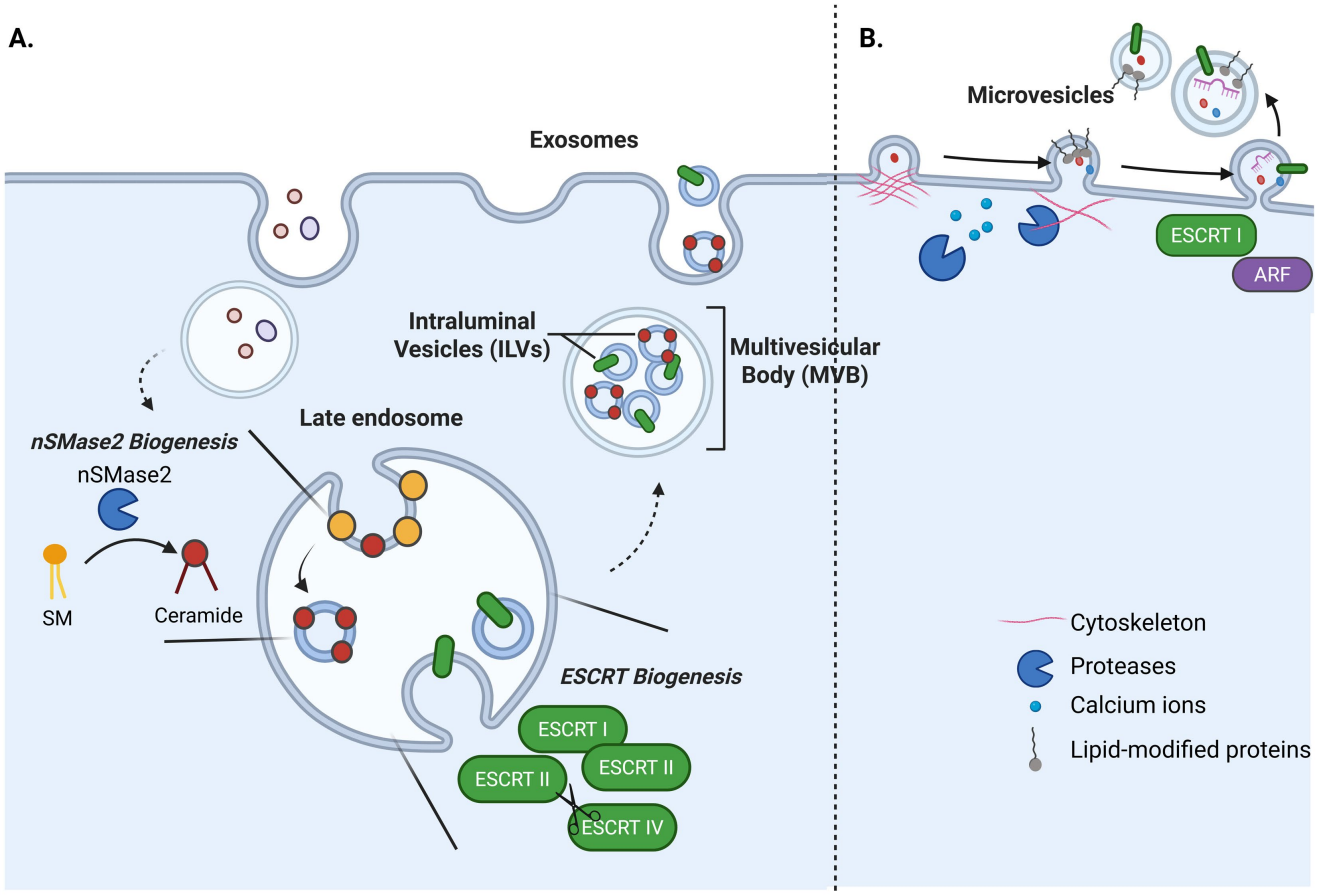
**(A)** In exosome biogenesis, ESCRT-dependent and ESCRT-independent pathways actively invert the endosomal membrane to form intraluminal vesicles (ILVs). An endosome laden with ILVs, also termed a multivesicular body (MVB), then merges with the plasma membrane where ILVs are released as exosomes. **(B)** In microvesicle biogenesis, increased concentration of proteases and calcium ions at the plasma membrane dissolves cytoskeletal proteins. Lipid-anchor modifications on proteins initiates outward budding of the plasma membrane, and cargo is sorted into membrane buds. ESCRT1 and ARF allow for the final pinching of the plasma membrane to release microvesicles into the extracellular space.

The most commonly studied EVs include exosomes, which are formed through the endolysosomal pathway, and microvesicles (also termed ectosomes) that bud from the plasma membrane. Exosome biogenesis begins when the membrane of late endosomes curve inward to form small vesicles in the endosome lumen, termed intraluminal vesicles (ILVs) (Figure 2A) (Gurung et al., 2021). Due to their appearance in electron microscopy, late endosomes containing ILVs are termed multivesicular bodies (MVBs). After ILV formation, an MVB fuses with the plasma membrane wherein the ILVs are released into the extracellular space as exosomes. Exosome biogenesis can be driven by a family of proteins known as the endosomal sorting complex required for transport (ESCRT). ESCRT-independent pathways have also been observed, such as the conversion of sphingomyelin to ceramide by the neutral sphingomyelinase 2 (nSMase2) protein (Trajkovic et al., 2008).

Microvesicles are formed by outward budding of the plasma membrane (Figure 2B). Microvesicle biogenesis pathways involve complex rearrangement of both cytoskeletal and membrane components, facilitated in part by a local increase in calcium ions and proteolytic enzymes that disassemble the cytoskeleton (Kalra et al., 2016). Membrane curvature is facilitated by lipid-anchor modifications on proteins (e.g., myristylation or palmitoylation), and the GTPase ADP-ribosylation factor (ARF) is required for secretion (Yu et al., 2024). Some proteins involved in exosome biogenesis are also implicated in microvesicle biogenesis, such as TSG101 and ALIX. Although they overlap in size, it is generally thought that exosomes are smaller (50–120 nm) than microvesicles (70–200 m).

One of the most interesting aspects of EVs is their molecular cargo, such as proteins, miRNAs, and lipids, which can be enriched disproportionate to what is found in their parent cell (Jeppesen et al., 2019; Couch et al., 2021; Gurunathan et al., 2021). This suggests that EVs are mechanistically packaged with specific molecules, either to remove them from the parent cell or transfer them to a recipient cell. Whether EV cargo is randomly integrated, intentionally curated, or some mix of both, is unknown. Yet in either case, EV cargo offers useful insight into the state of the parent cell. Cellular perturbations, either through disease or experimental manipulation, are known to alter EV cargo. Further, the functional effects that EVs have on recipient cells can be altered based on changes to the parent cell. This suggests that, random or not, EV cargo has important implications for parent cell biology and cell-to-cell communication, especially in the context of disease.

## 4 Current understanding of EVs in NPC

At the time of this writing, we are aware of only 18 peer-reviewed publications that have directly examined EVs in the context of NPC (Table 1). Sixteen of these studies analyzed EVs in response to *NPC1* mutations, while only two examined *NPC2* mutations. Four studies were performed *in vivo,* using either the NPC1^m1N^/J or NPC1^nmf174^/J mouse. The remaining experiments were performed using *in vitro* models, including immortalized cell lines, primary murine cells, or patient-derived fibroblasts. Many studies used genetic models, while others treated cells with U18666A (U18), a small molecule inhibitor that specifically inactivates the NPC1 sterol-sensing domain and reproduces NPC endolysosomal cholesterol accumulation (Lu et al., 2015). Three studies used cells that mimic neurological phenotypes, including oligodendrocyte precursors (Oli-neu), rat primary astrocytes, and rat primary aged cortical neurons. Only four examined EVs in NPC human samples, each of which relied on fibroblasts derived from a single patient. Given the high degree of heterogeneity among NPC patients, studies on larger cohorts of NPC human samples are needed.

**Table 1:** Peer-reviewed studies of EVs in NPC disease models.

Paper	Experimental system	NPC EV profile (concentration/cargo)	Mechanistic findings
*In vitro* studies			
**Strauss et al. (2010)**	NPC1 fibroblast (R934X/P1007A); Oli-neu cells+U18; *NPC1* siRNA	Increased EV release; Increased EV cholesterol	Cholesterol accumulation leads to increased EV release
**Ilnytska et al. (2021)**	NPC1 fibroblast (P237S/I1061T)		NPC1 phenotype ameliorated via release of cholesterol-laden EVs
**Juhl et al. (2021)** *	NPC2 fibroblast (GM18455)		NPC2 transports cholesterol from EL allows to allow efflux via EVs
**Wüstner et al. (2023)** *	NPC2 fibroblast (GM18455)		NPC2 is required for EV-driven sterol export
Canfrán-Duque et al. (2014)	HEPG2 and THP-1 cells + U18	No change to EV release	
Cashikar and Hanson (2019)	T-Rex-293 cells + U18	No change to EV release	
Feltes et al. (2020)	U2OS cells with *NPC1* KO		EVs do not mediate HPBCD-driven cholesterol egress from NPC cells
Albacete-Albacete et al. (2020)	Mouse embryonic fibroblasts + U18	Increased EV release	
Costafreda et al. (2020)	*NPC1* KO Hu7 cells		NPC1 facilitates HAV EV cargo uptake
Wu et al. (2021)	Rat primary astrocytes + U18	Decreased EV release	
Lu et al. (2022)	K562 cells+ U18		Deletion of ESCRT proteins increases cholesterol in NPC KO
Elgner et al. (2016)	Hu7.5 cells + U18		U18 inhibits exosome-mediated release of HCV viral particles
Guix et al. (2021)	Aged rat cortical neurons + U18	Increased EV release	Decreased NPC1 expression and increased cholesterol drive increased EV release
Palmulli et al. (2024)	HeLa and MNT-1 cells + U18		CD63 and NPC1 sort cholesterol into ILVs for release as small EVs
*In vivo* studies			
Chen et al. (2010)	Cells from BALB/*cnpc*^nih^		HPBCD treatment increases endolysosomal EV release in a calcium-dependent manner
Van Hoecke et al. (2021a)	NPC1^m1N^/J mouse (choroid plexus)	Decreased CD81/CD9 EVs release; Larger EVs	NPC choroid plexus EVs are cytotoxic
Van Hoecke et al. (2021b)	NPC1^m1N^/J mouse		WT human MSC EVs ameliorate NPC pathology
Soto-Huelin et al. (2023)	NPC1^nmf174^/J mouse	Increased hex-ceramide and LPC lipid content	NPC1 phenotype ameliorated by stimulating increased EV release

Bolded text indicates studies that used human patient samples; Stars indicate studies on NPC2 mutations. EL, endolysosomes; PM, plasma membrane; HCV, Hepatitis C Virus; HAV, Hepatitis A Virus.

The existing studies on EVs in NPC have primarily focused on the mechanistic role of EVs in NPC pathways, or on how NPC disease affects EV production. It is important to consider that protocols for EV enrichment and analysis vary widely between publications, and results must be interpreted within their methodological context. Despite these limitations, there is growing evidence that the NPC proteins play direct roles in EV biology; that EV concentration and cargo is altered by NPC pathology; and that EV packaging and release may be one method by which NPC attempts to remove cholesterol from endolysosomes in cells. To further understand how EVs contribute to or are affected by NPC cellular disruptions, these studies must be carefully considered and built upon.

### 4.1 NPC proteins in EV uptake and release

The NPC proteins have been directly implicated in both uptake and release of EVs. In immortalized cell lines, NPC1 was proven essential for the endocytosis of EVs from Hepatitis A-infected cells (Costafreda et al., 2020), as well as for the release of endosomal EVs containing Hepatitis C viral particles (Elgner et al., 2016) and cholesterol (Palmulli et al., 2024). In patient-derived fibroblasts, NPC2 can facilitate the release of cholesterol-rich ILVs as exosomes (Wüstner et al., 2023), and the shedding of microvesicles from the plasma membrane (Juhl et al., 2021). These studies suggest that mutations in *NPC1* or *NPC2* may directly impact EV release and uptake, both at the plasma membrane and within endosomes.

### 4.2 EV concentrations in NPC samples

There is conflicting evidence as to whether NPC samples contain increased (Strauss et al., 2010; Albacete-Albacete et al., 2020; Guix et al., 2021), decreased, or no change (Canfrán-Duque et al., 2014; Cashikar and Hanson, 2019) in EV concentrations relative to control samples. A study by Strauss et al. (2010) reported that NPC pathology, modeled through siRNA knockdown of *NPC1*, U18 treatment, or cholesterol-loading cells, led to increased release of EVs in cell culture. They further showed that following *NPC1* knockdown, EVs contained higher concentrations of cholesterol and were released in a Flotillin-1 dependent manner, which they replicated in patient-derived fibroblasts. Their results were supported by Albacete-Albacete et al. (2020), who found that treating mouse embryonic fibroblasts with U18 increased EV release. However, other authors have found that treating the immortalized cell lines HEK293 (Cashikar and Hanson, 2019), HepG2 (Canfrán-Duque et al., 2014), and THP-1 (Canfrán-Duque et al., 2014) with U18 does not alter EV concentration. These contrasting results may indicate that the relationship between NPC1 inhibition and EV concentration is cell-type dependent.

As NPC is characterized by both peripheral and neurological defects, studies have also examined EVs in CNS models of NPC. In the choroid plexus of NPC^−/−^ mice, Van Hoecke et al. (2021b) found significantly fewer and larger CD81- or CD9-positive EVs as compared to controls, suggesting a possible change in EV subpopulations. Guix et al. (2021) showed that rat cortical neurons increase EV release following exposure to U18 and used genetic manipulations to demonstrate that NPC1 regulates ILV production. In contrast, rat astrocytes decreased EV release following U18 treatment, suggesting that neurons and astrocytes respond to NPC1 inhibition differently (Wu et al., 2021).

Complicating the comparison of these studies is the fact that the experiments utilized different *in vitro* and *in vivo* models of NPC, as well as different EV enrichment and analysis techniques. Yet taken together, they suggest that the effect of NPC pathology on EV concentration likely varies by cell type, animal model, and the extent to which the NPC1 protein is inhibited. Importantly, the only study to examine EV concentration in NPC patients used a single fibroblast cell line from one person (Strauss et al., 2010). Given the large heterogeneity among NPC patients in terms of genetics and disease progression, repeated analysis in a larger patient cohorts is critical. Additionally, a better understanding of how EV concentrations are altered in visceral versus CNS tissues could reveal important biology behind the neurodegenerative impacts of *NPC1* mutations.

### 4.3 The molecular cargo of NPC EVs

To fully understand the role that EVs play in NPC, careful characterization of NPC EV cargo is needed. Two studies have reported that EVs from NPC sources (NPC-null mice or U18-treated astrocytes) cause significant cell death and stress signaling in recipient cells, suggesting that NPC EVs are enriched in cytotoxic material (Van Hoecke et al., 2021b; Wu et al., 2021). Wu et al. (2021) linked this cytotoxicity to the increased amyloid precursor protein (APP) and Aβ peptides found in U18-treated astrocytic EVs. These markers are highly relevant to the development of AD, which shares many similarities with NPC (Malnar et al., 2014). The authors also noted significantly decreased levels of Lamp1 and LC3 expression in EVs from U18-treated cells, implicating EVs in autophagy disruptions previously observed in NPC (Pacheco and Lieberman, 2008).

Understanding how lipid dysregulation in *NPC1*-mutated cells affects EV lipid content is also of great interest. As described previously, Strauss et al. found increased cholesterol content in EVs from an NPC patient cell line (Strauss et al., 2010). Another study found a general increase in lipid content of EVs from NPC^−/−^ mouse brain compared to controls, including significant enrichment of lysosphosphatidylcholine (LPC) and hexosylceramide. LPC has been previously implicated in NPC and has been proposed as a potential biomarker for patient response to candidate drug treatments (Mishra et al., 2024), while ceramide lipids are linked to the progressive death of Purkinje neurons in NPC patients (Tobias et al., 2019; Wanikawa et al., 2020). Importantly, exosomes produced by the nSMAse2 pathway are ceremide-rich (Figure 2A) (Trajkovic et al., 2008) and the accumulation of this exosome subtype has been implicated in the progression of AD. Yet whether ceramide-rich, nSMase2-derived exosomes also contribute to NPC pathology has not been tested (Dinkins et al., 2014; Dinkins et al., 2016).

Collectively, these studies emphasize that EVs from NPC sources are enriched in proteins and lipids that are highly relevant to NPC disease pathology. An important knowledge gap is how EV miRNA content is altered following *NPC1/2* mutations, which has not yet been studied. This is despite the fact that miRNAs are known to be altered in NPC (Ozsait et al., 2010; Pergande et al., 2019) and that EV miRNAs are putative biomarkers in other neurodegenerative diseases. Additionally, most analyses of NPC EV cargo have been performed in mouse models, and very little is known about EVs in NPC patient samples. As with other neurodegenerative diseases and lysosomal storage disorders, further research on EV cargo could inform on the cellular mechanisms of NPC pathology and possibly allow EVs to be used as NPC biomarkers (Vella et al., 2016; Tancini et al., 2019).

### 4.4 EVs and NPC therapeutics

NPC therapeutics often aim to lower endolysosomal cholesterol, either by redistributing it to other cellular locations or removing it from the cell entirely. Some researchers have hypothesized that the removal of cellular cholesterol in NPC-mutated cells could be facilitated by EVs. For example, treating NPC^−/−^ mice with the candidate treatment ellagic acid dramatically increased EV release while reducing levels of cellular cholesterol and other lipids (Soto-Huelin et al., 2023). Similarly, treating NPC patient-derived fibroblasts with phosphatidylglycerol substantially reduced cellular cholesterol by exporting it via EVs (Ilnytska et al., 2021). Conversely, inhibiting EV release in NPC cells has damaging effects. In a patient cell line, inhibiting exosome release with GW4869 (a drug that blocks nSMase2-driven exosome biogenesis) caused significant accumulation of cholesterol-rich vesicles at the plasma membrane (Ilnytska et al., 2021). A CRISPR screen performed in an immortalized cell line under U18 treatment showed that deletion of any of the ESCRT proteins increased cellular cholesterol (Lu et al., 2022), in agreement with a previous study showing that knockdown of ESCRT-0 causes NPC-like cholesterol accumulation (Du et al., 2012). Together, these studies suggest that EVs play a beneficial role in NPC, possibly by removing endolysosomal cholesterol. However, a direct therapeutic benefit of EV-mediated cholesterol removal in NPC cells has not yet been demonstrated. Recently, Van Hoecke et al. (2021a) demonstrated that EVs from mesenchymal stem cells alleviated inflammation in peripheral organs and brain of NPC1^−/−^ mice, suggesting EVs themselves may be a promising NPC therapeutic.

In 2024, the U.S. Food and Drug Administration approved the first two drugs for NPC: Miplyffa (arimoclomol) was approved in combination with the enzyme inhibitor miglustat to treat NPC neurological symptoms in patients 2 years of age and older; and Aqneursa (levacetylleucine) was approved to treat neurological symptoms in NPC patients weighing at least 15 kilograms (Jan et al., 2025). While the impact of these medications on EVs has not been directly investigated, each acts on pathways relevant to EV biology. Arimoclomol activates heat-shock proteins commonly carried by EVs (Kowal et al., 2016); miglustat inhibits the synthesis of glycosphingolipids, a common component of EV lipid membranes (Phuyal et al., 2014; Skotland et al., 2019); and levacetylleucine stabilizes mitochondrial function, which can impact EV formation and release (Picca et al., 2020; Bonora et al., 2024). Whether EVs are involved in the therapeutic mechanisms of these medications remains to be tested.

## 5 Discussion

Current studies suggest that EVs are altered in NPC, and that these changes are relevant to NPC pathology. Though limited, the existing literature raises several interesting questions about the relationship between NPC and EVs. The conflicting evidence on EV concentration suggests that the ways in which the NPC proteins affect EV formation and release are complex, and may be dependent on cell type, model system, and the subspecies of EVs analyzed (exosomes, microvesicles, etc.). Of particular relevance is whether there are CNS-specific changes to EVs in NPC which may help explain the neurodegenerative aspects of the disease. It is also interesting to note that loss of NPC1 protein function generates EVs that carry protein and lipid content related to NPC disease pathology. This could suggest that EVs are a useful method for NPC mutant cells to export harmful material; however, as EVs are often taken up by recipient cells, this may also implicate EVs in the spread pathologic molecules throughout the body.

Critical gaps in the current literature also limit our understanding of EVs in NPC. For example, there is not yet any information on how EV miRNAs are altered in NPC, and full proteomic analyses have not yet been conducted. As with other aspects of NPC the majority of studies have focused on the impact of *NPC1* loss, and although the NPC2 protein plays known roles in EV biogenesis, no studies to date have examined how *NPC2* mutations affect EV concentration or cargo. Additionally, the lack of studies in human samples makes it difficult to determine how EV alterations impact NPC patients. As no studies to-date have examined EVs collected from patient biofluids, it is currently unknown how EVs from differently impacted organs in NPC—such as the liver, brain, or inner ear—may contribute to disease pathology. Patient heterogeneity must also be considered. Many of the current EV NPC studies utilized *in vitro* systems with full knockdown of the NPC1 protein, either by genetic alterations or drug treatment. However, the degree of protein dysfunction in NPC patients varies widely, and full loss of NPC1 or NPC2 protein expression is less common in pediatric or adult patients. How EVs are affected by incomplete loss *NPC1* or *NPC2* is unknown, and it is possible that this too varies by individual.

Finally, the molecular connection between loss of NPC1/NPC2 protein function and EV alterations is also currently unknown. Some research suggests that NPC1 and NPC2 play direct roles in EV release and uptake yet is also possible that EVs are impacted by other cellular changes caused by *NPC1/NPC2* mutations. For example, endolysosomal dysfunction, oxidative stress, and altered autophagic flux are known to occur in NPC (Wheeler and Sillence, 2020), and evidence from other disease states suggest that these cellular perturbations impact EV biology (Eitan et al., 2016; Chiaradia et al., 2021; Ai et al., 2024). Conversely, discoveries about the impact of NPC cellular pathology on EVs may shed light on other diseases that share similar mechanisms. Indeed, outside of NPC, there is a growing appreciation of how endolysosomal injury affects EV production. Thus, further research into NPC and EVs will contribute to our growing understanding of EV cellular biology and reveal novel information about a childhood disease that currently has no cure.

## Footnotes

1
https://www.proteinatlas.org/ENSG00000198910-L1CAM/tissue
